# Comparing the Effectiveness of Atropine Sulfate and Acacia Catechu in Reducing Salivary Secretion During Dental Procedures: Protocol for a Randomized Controlled Trial

**DOI:** 10.2196/78119

**Published:** 2026-11-13

**Authors:** Mrunmayee Pophali, Mithilesh Dhamande, Surekha Dubey, Aashish Gupta

**Affiliations:** 1 Department of Prosthodontics, Sharad Pawar Dental College and Hospital, Datta Meghe Institute of Medical Sciences, Room No. 202, PH7G+P93 Datta Meghe Institute of Medical Sciences Campus, Sawangi Meghe, Wardha, 442001, India, 1 8600251446

**Keywords:** sialorrhea, atropine sulfate, sublingual eye drops, *Acacia catechu*, salivary flow rate

## Abstract

**Background:**

Sialorrhea, or excessive salivation, can be a major problem during a number of dental operations. It can impair vision, increase chair time, and perhaps jeopardize the effectiveness of treatments. To treat this illness, pharmacological treatments such as atropine sulfate are frequently used. However, there has not been much research done on natural substitutes like *Acacia catechu* for this function.

**Objective:**

The aim of this study is to compare and evaluate the effectiveness of atropine sulfate and *Acacia catechu* in reducing salivary secretion in patients undergoing restorative dental procedures.

**Methods:**

A randomized controlled trial will be conducted involving 160 participants undergoing restorative dental treatments. Participants will be randomly assigned to receive either atropine sulfate or *Acacia catechu*. Salivary flow will be measured preintervention and postintervention. The primary statistical analysis will use analysis of covariance, with postintervention salivary flow as the dependent variable and baseline salivary flow as a covariate. If a placebo arm is included, group differences will be analyzed using 1-way ANOVA followed by Tukey post hoc tests. Paired *t* tests will be used only for within-group pre-post comparisons.

**Results:**

Ethical approval was obtained in March 2025. Participant recruitment is scheduled to begin in June 2026 and is expected to be completed by December 2026, with final data collection anticipated by February 2027. It is anticipated that both interventions will reduce salivary flow, with atropine sulfate demonstrating greater efficacy but more side effects, and *Acacia catechu* offering a better side-effect profile and higher patient acceptability.

**Conclusions:**

The trial will provide comparative evidence on the efficacy and safety of a pharmacological and a natural anticholinergic agent. If *Acacia catechu* proves effective, it may offer a viable, better-tolerated alternative for salivary control in clinical dental settings.

## Introduction

### Background

Salivary secretion is essential for oral health and is mainly regulated by parasympathetic neural control via facial and glossopharyngeal nerves. When these nerves are activated, they release neurotransmitters like acetylcholine. Controlling saliva is essential in prosthodontic procedures to maintain a dry and clean environment, which leads to better precision, comfort, and long-term success of prosthetic treatments. However, excessive salivation (sialorrhea) during oral surgical procedures can pose challenges to procedures such as taking impressions, lower arch surgery, and placing restorations. During crown preparation, controlling the amount of saliva is essential to maintain a dry field. It can interfere with impression-making, bonding procedures, and proper fitting of the crown.

The white crystalline alkaloid atropine was originally synthesized from *Atropa belladonna*. It inhibits the normal functioning of the M-choline receptor. Salivation is caused by the activation of muscarinic subtype 3 receptors [1,2]. The muscarinic receptors of the salivary glands are bound by the active neurotransmitter acetylcholine. Thus, it is well known that atropine and other cholinergic muscarinic receptor antagonists can be used to treat hypersecretion by the gland [3].

For oral administration of atropine in adults, a pharmacokinetics investigation was done by Beermann et al [4] using atropine that was radiolabelled. The absorption of atropine via the oral route in children was examined in two investigations [5,6]. Numerous alternative regimen tactics have been documented in the literature to specify atropine dosages and frequencies based on the patient’s clinical status. Atropine sulfate delivered sublingually has several benefits, including ease of administration and fast mucosal membrane absorption. It is affordable, reversible, and does not require any particular skills to use [7]. Additionally, it is often more accepted by patients than invasive methods like injections or other tools. Low dosages of atropine given orally or sublingually may help these patients. Atropine side effects include nausea, vomiting, dry mouth, impaired vision, and tachycardia. There is limited research on the safety and pharmacokinetics of atropine given sublingually. Notwithstanding the low dosages used in this application, little is known about atropine’s systemic absorption when taken sublingually, necessitating extra caution when it comes to the possibility of side effects.

In contrast, natural plant-based alternatives with astringent properties are gaining attention in modern dentistry for their therapeutic potential and lower risk profiles.

For many years, Ayurveda has used the deciduous *Acacia catechu* (L.f.) Willd. tree, which belongs to the Fabaceae family, to cure and prevent a wide range of illnesses and conditions [8]. Several parts of *Acacia catechu* have an exceptionally high concentration of flavonoids, tannins, and phenolic compounds, such as procyanidin, quercetin, catechin, epicatechin, epigallocatechin, and taxifolin [9]. These active ingredients, including catechin and epicatechin, have important anti-inflammatory and antioxidant properties; tannins are thought to cause the astringent effects on the body [10], reducing secretions like saliva. These properties suggest that *Acacia catechu* may help reduce salivary secretion through local action on the oral mucosa, without the systemic effects seen with pharmacological agents like atropine. By assessing both clinical outcomes and patient comfort, this study seeks to determine whether *Acacia catechu* may serve as a viable, natural alternative to conventional anticholinergic agents in dental practice.

### Aim

The aim of this study is to compare and evaluate the effectiveness of atropine sulfate and *Acacia catechu* in reducing salivary secretion in patients undergoing restorative dental procedures.

### Objective

The objectives of the study are to assess the amount of salivary secretion before and after intervention with atropine sulfate and *Acacia catechu* and to compare the efficacy of both agents. Additionally, the study aims to evaluate patient acceptability and tolerance for each intervention.

## Methods

### Trial Design

This is a parallel-group, randomized, double-blind, controlled trial with 1:1 allocation. A total of 160 participants undergoing restorative dental procedures will be recruited and randomized into 2 equal groups to receive either atropine sulfate or *Acacia catechu*.

Group A participants will receive 1% w/v atropine sulfate drops (commercial preparation) sublingually, at a dose of 0.01 mg/kg, 30 minutes prior to the dental procedure.

Group B participants will receive an aqueous extract of *Acacia catechu* prepared from dried heartwood. To prepare the extract, 100 g of powdered *Acacia catechu* will be boiled in 1000 mL of distilled water until reduced to one-third of its original volume. The decoction will be filtered, cooled, diluted (2‐5% w/v), and stored under sterile conditions at 4°C until use.

The *Acacia catechu* extract w/v) will be administered sublingually using a sterile dropper, at a dose of 1 mL/kg, 30 minutes before the dental procedure. Participants will hold the extract under the tongue for about 2 minutes to allow mucosal absorption before swallowing.

All participants will be seated comfortably in the dental chair and will be instructed to rinse with plain water before baseline measurements. Unstimulated saliva will be collected over a 5-minute period using the spit method into a preweighed, sterile container. Baseline heart rate and blood pressure will be recorded using a digital monitor.

To maintain blinding, participants and outcome assessors will not be informed about which intervention group the participant was assigned to. The preparation of both solutions will be handled by an unblinded third party. The principle of intent-to-treat will be applied, and all participants will be required to provide written informed consent before enrollment. Before the study begins, the patient’s complete medical, dental, and demographic history will be recorded. The data will be tabulated and statistically evaluated using SPSS (version 21; IBM Corp) software*,* while the student *t* test will be used for intergroup analysis. The schedule of enrollment, interventions, and assessments is outlined in Table 1, following SPIRIT (Standard Protocol Items: Recommendations for Interventional Trials) guidelines.

**Table 1.** Timeline of participant enrollment, allocation, intervention delivery (atropine sulfate or *Acacia catechu*), and data collection points for outcomes including salivary flow rate, adverse events, patient comfort, onset of action, and duration of effect, based on the SPIRIT (Standard Protocol Items: Recommendations for Interventional Trials) 2013 guidelines.

Time point/study period	Enrollment	Allocation	Preintervention	Intervention	Postintervention
Informed consent	X				
Eligibility screening	X				
Randomization		X			
Salivary flow assessment			X		X
Atropine group				X (administered)	
*Acacia catechu* group				X (administered)	
Adverse event monitoring					X

### Participants

#### Inclusion Criteria

The inclusion criteria for the study are as follows:

1. Patients going through restorative dental procedures.
2. Patients aged between 18 and 65 years.
3. Patients willing to participate with informed consent.

#### Exclusion Criteria

The exclusion criteria are as follows:

1. Patients aged below 18 years.
2. Patients with known allergies to atropine or *Acacia catechu.*
3. Patients with preexisting xerostomia (dry mouth).
4. Patients on medications affecting salivary secretion.
5. Pregnant or lactating women.

### Outcome Measures

#### Primary Outcome

The primary outcome of the study will be the change in unstimulated salivary flow rate (mL/min). Unstimulated whole saliva will be collected using the standardized spit method at baseline (preintervention) and at 30 minutes postintervention. The salivary flow rate will be calculated by dividing the volume of saliva collected by the duration of collection (5 min). The 30-minute time point was selected because atropine’s anticholinergic action and *Acacia catechu*’s astringent effect both reach measurable changes within 20‐40 minutes.

#### Secondary Outcomes

The following secondary outcomes will also be assessed.

1. Incidence and severity of adverse effects. A standardized side-effect checklist will be used to record known atropine-related anticholinergic effects. Participants will be evaluated at baseline and 30 minutes postintervention for dry mouth, blurred vision, tachycardia or palpitations, dizziness, difficulty swallowing, and facial flushing, along with any other adverse events recorded in a free-text field. Each symptom will be rated on a 4-point severity scale: 0 (none), 1 (mild), 2 (moderate), and 3 (severe). Heart rate and blood pressure will also be recorded at both time points.
2. Patient comfort and acceptability. Patient acceptability will be assessed 30 minutes postintervention using two validated measures: (1) Taste acceptability will be evaluated using a 100 mm visual analog scale (VAS), where 0 indicates extremely unpleasant and 100 indicates extremely pleasant. (2) Overall satisfaction will be evaluated using a separate 100 mm VAS, where 0 indicates not satisfied at all and 100 indicates extremely satisfied. Ease of use will be assessed using a 5-point Likert scale (1=very difficult, 2=difficult, 3=neutral, 4=easy, and 5=very easy). All patient-reported outcomes will be completed privately to avoid bias.
3. Clinician-reported outcome (dentist assessment). The operating dentist will provide a blinded assessment of the intraoral field of view during the procedure using a standardized 5-point rating scale, where 1=poor, 2=fair, 3=adequate, 4=good, and 5=excellent. The dentist will also record whether salivary control was clinically acceptable (yes/no).
4. Onset of action. The onset of action will be defined as the time interval from the administration of the intervention to the first observed reduction in salivation, which will be determined either subjectively (patient-reported) or objectively (clinician-assessed).
5. Duration of effect. The duration of the salivary suppression effect will be measured from the time of onset until the return to baseline salivary flow rate, as recorded in follow-up assessments.

### Sample Size

#### Sample Size Formula for Designing Clinical Research

The sample size was estimated based on data from a prior study comparing atropine and glycopyrrolate [11]. The notation for the formulas is as follows:


$$n_{1}=\frac{\left(\sigma_{1}^{2}+\frac{\sigma_{2}^{2}}{k}\right){\left(Z_{1-\alpha /2}+Z_{1-\beta }\right)}^{2}}{\Delta^{2}}$$

- *n₁*=Group 1 sample size
- *n₂*=Group 2 sample size
- *σ₁*=Group 1 standard deviation
- *σ₂*=Group 2 standard deviation
- *Δ*=difference in means of the group
- *κ*=ratio=*n₂/n₁*
- *Z₁-α/2*=two-sided *Z* value (eg, *Z*=1.96 for 95% CI)
- *Z₁-β*=power

Mean difference in saliva in atropine group=0.94000

Mean difference in saliva glycopyrrolate group=1.0667

σ1=SD of saliva in atropine group=0.38508

σ2=SD of saliva glycopyrrolate group=0.41173

For detecting mean difference of 0.1267, ie, ∆=1.0667‐0.9400=0.1267

K=1


$$N=\frac{(0.38508^{*}0.38508+0.41173)(1.96+0.84{)}^{2}}{0.1267^{*}0.1267}$$

=155.21=160 patients needed in study

Power of the test: 80%

Level of significance: 5% (95% CI)

### Randomization and Blinding

#### Randomization

Patients will be randomized into 2 groups using computer-generated block randomization.

#### Blinding

This study will be conducted as a double-blind, randomized controlled trial to minimize bias. Participants and outcome assessors will be blinded to the treatment allocation.

To accomplish blinding despite the difference in interventions (atropine sulfate drops vs *Acacia catechu* extract), matching placebos will be used as follows:

Group A will receive the active *Acacia catechu* extract along with a placebo atropine preparation (an inert solution mimicking atropine drops in appearance and taste).

Group B will receive the active atropine sulfate drops along with a placebo *Acacia catechu* preparation (an inert solution matching the herbal extract’s color and viscosity).

These interventions and placebos will be prepared and dispensed by a third-party pharmacist or staff member not involved in participant assessment or interactions. The interventionist administering treatments will be blinded to group allocation, handling only coded bottles labeled by the third party. If preparation of matching placebos is not feasible, this will be justified explicitly in the protocol, and measures will be documented to ensure at least single-blindness of the outcome assessor. Participants will be instructed not to discuss sensations or side effects with outcome assessors, and assessors will be trained to use objective salivary flow measures to limit bias.

### Data Collection and Management

#### Data Collection Procedures

Data will be collected at 2 time points for each participant.

#### Baseline (Preintervention)

At baseline, we will collect demographics, medical history, baseline salivary flow, and vital signs (heart rate, blood pressure).

#### Postintervention (Within 1 Hour)

Postintervention, we will collect salivary flow, adverse events, patient-reported comfort (VAS), operator ease-of-use score, time-to-onset, and duration of the antisialogogue effect. Saliva will be collected by the spit method in a preweighed container for a 5-minute unstimulated period. Vital signs will be recorded using a digital monitor. Adverse events will be documented using a structured checklist.

### Data Management

All data will be initially recorded on paper case report forms. Data will be digitized using Microsoft Excel (Microsoft Corp) and stored on a password-protected institutional computer system. Each participant will be assigned a unique ID to maintain confidentiality. A double-entry method will be used to minimize data entry errors. Regular audits will be conducted to ensure accuracy, completeness, and compliance.

### Statistical Analysis Plan

To assess variation in salivary secretion among the study groups, the data will first be summarized using descriptive statistics, including mean, standard deviation, and range. Categorical variables such as the presence of adverse effects will be compared using the *χ*² test.

For the primary analysis, an analysis of covariance will be performed, with postintervention unstimulated salivary flow as the dependent variable and baseline salivary flow as the covariate. This method provides an adjusted and robust comparison of treatment effects.

If the study includes three arms (atropine, *Acacia catechu*, and placebo), overall group differences will be evaluated using 1-way ANOVA, followed by a Tukey post hoc test to identify pairwise differences.

Within-group (preintervention vs postintervention) changes will be analyzed using paired *t* tests, whereas comparisons between two independent groups (eg, atropine vs *Acacia*) will use unpaired *t* tests only for exploratory purposes.

All analyses will be performed using SPSS (version 27.0; IBM Corp) and GraphPad Prism (version 7.0; GraphPad Software). Statistical significance will be set at *P*<.05.

### Ethical Considerations

The institutional review board has granted ethical approval [DMIHER(DU)/IEC/2025/549]. There will be safety monitoring for adverse drug reactions. Informed consent will be obtained from all participants. The study will follow strict guidelines for the ethical handling of patients.

### Dissemination

Upon completion of the trial, results will be submitted to peer-reviewed journals and presented at scientific conferences. Participants will be informed of key findings upon request.

## Results

Ethical approval for the study was obtained from the DMIHER (DU) (approval number DMIHER(DU)/IEC/2025/549) in March 2025. The study has been registered with the Clinical Trials Registry – India (CTRI/2025/06/089468). As of April 2026, the study had not yet initiated participant recruitment, and no participants had been enrolled. No outcome data had been collected or analyzed. Participant recruitment is scheduled to begin in June 2026. The planned sample size is 160. Recruitment is expected to be completed by December 2026, with final data collection anticipated by February 2027. Data analysis will follow completion of data collection.

## Discussion

For the majority of contemporary intraoral operations, effective saliva control and a well-separated operating space are prerequisites. This protocol draws upon prior research supporting the use of 1% atropine sulfate as an effective anticholinergic agent. The planned method of sublingual administration is supported by studies demonstrating its pharmacokinetic advantages. In a brief paper published in 2002, Hyson et al [12] justified the use of ocular atropine drops by pointing out that they are readily available, inexpensive, and do not require specific knowledge to administer. Meningaud et al [13] made a similar case, citing its reversible impact as an extra advantage. The same justification for atropine administration via ocular drops has also been used in other research [14-16].

In this planned study, the medication will be administered sublingually to ensure rapid absorption and avoid first-pass metabolism, which is inevitable when taking it orally. Due to enhanced perfusion, this ensures that the medicine enters the bloodstream more quickly than through oral or intramuscular methods and enhances absorption [7]. Additionally, in prior research where the safety of ophthalmic atropine drops has been demonstrated, including in the pediatric population, this method has been the most frequently used for these drops [17].

*Acacia catechu*, a traditional herbal remedy, contains bioactive compounds such as catechin and epicatechin, which possess astringent properties. These compounds can cause protein precipitation and mucosal contraction, leading to reduced salivary secretion. An in vitro study by Pandey [18]found a significant decrease in the colony-forming unit count per milliliter of saliva after the application of *Acacia catechu* paste, indicating a reduction in microbial load and potentially salivary flow. Atropine is contraindicated in patients with conditions such as glaucoma, prostatic hypertrophy, and certain cardiac arrhythmias due to its anticholinergic effects. In contrast, *Acacia catechu* is generally considered safe when used appropriately, with minimal risk of systemic side effects. However, allergic reactions to plant-based products, though rare, should be considered.

The present study aims to compare the effectiveness of atropine sulfate and *Acacia catechu* in reducing salivary secretion during dental procedures through an in vivo assessment, evaluating both clinical efficacy and patient tolerance.

The study will assess the following:

1. The efficacy of atropine sulfate and *Acacia catechu* in reducing salivary secretion during dental procedures.
2. The onset of action of both agents (time taken to begin reducing salivation).
3. The duration of the effect (how long the salivary suppression lasts).
4. The incidence and severity of side effects associated with each agent.
5. Patient acceptability and subjective experience (eg, taste, comfort, dryness).

## Abbreviations

SPIRIT: Standard Protocol Items: Recommendations for Interventional Trials
VAS: visual analog scale
